# A socio-ecological approach to inclusive physical education in China: A systematic review

**DOI:** 10.3389/fpubh.2022.902791

**Published:** 2022-07-28

**Authors:** Xiao Liang, Minghui Li, Yandan Wu, Xueping Wu, Xiaohui Hou, Cindy Hui-Ping Sit

**Affiliations:** ^1^Department of Sports Science and Physical Education, The Chinese University of Hong Kong, Hong Kong, China; ^2^School of Physical Education and Sports Science, Fujian Normal University, Fuzhou, China; ^3^School of Physical Education and Sport Training, Shanghai University of Sport, Shanghai, China; ^4^Department of Sport and Health, Guangzhou Sport University, Guangzhou, China

**Keywords:** inclusive physical education, students with special education needs, systematic review, China, socio-ecologic model

## Abstract

**Background:**

Since the implementation of inclusive education in China, students with special education needs (SEN) have increasingly been integrating into mainstream schools, like physical education classes. However, inclusive physical education (IPE) in China has developed slowly, and gaps can be found in the knowledge of the factors that inhibit or promote the participation in IPE of students with SEN.

**Objectives:**

The purpose of this systematic review was to provide a comprehensive summary of the factors related to inclusion in IPE of students with SEN, by applying a socio-ecological model (SEM). Five databases were searched: ERIC, SPORTDiscus with Full Text, Education Full Text (H.W.Wilson), PsychINFO and CNKI in March 2022, to find studies that identify factors regarding IPE in China. Two researchers independently screened studies and summarized relevant data.

**Results:**

Fourteen studies were included in the detailed review. By applying the SEM, multi-level factors were identified, ranging from intrapersonal to societal levels that positively or negatively influenced IPE participation in students with SEN. This review indicates that multi-level factors affect the IPE participation of students with SEN in China.

**Conclusion:**

The findings will help assist educators and policymakers to develop effective IPE for Chinese students with SEN.

## Introduction

Inclusive education is an approach that aims to eliminate social exclusion, on the premise that education is a foundation for society. It has been accepted as a core education policy worldwide (1, 2). The concept of inclusive education was developed under the influence of the Salamanca Statement (3), indicating the fundamental idea of inclusive education is that “every child has the fundamental right to receive education, and must be given the opportunity to achieve and maintain an acceptable level of learning; those with special education needs (SEN) must have access to regular schools which should accommodate them within a child-centered pedagogy, capable of meeting these needs” (p. 3). Following the development of this educational philosophy, empirical studies have documented the benefits of inclusive education, such as improving the academic learning of students with and without SEN (4, 5), enhancing their social interactions (6) and helping them to achieve a more positive self-concept (7). Through the philosophy of inclusive education and followed by the relevant implementation of legislation and policies as well as the evidence-based research in this area, students with SEN are able to be well-educated in mainstream schools.

Inclusive physical education (IPE) has been promoted as a fundamental human right (8). Previous studies have highlighted the importance and benefits of IPE for students with SEN (9, 10). Three systematic reviews regarding IPE have examined the factors that affect their participation during IPE among students with SEN. Block and Obrusnikova (11) reviewed 38 relevant studies conducted between 1995 and 2005 and summarized six aspects influencing IPE: (a) support from peer tutors, teaching assistants and adapted PE specialists, (b) effects of typically developing (TD) peers, (c) attitudes and intentions of children without SEN, (d) social interactions, (e) academic learning time of students SEN during PE, and (f) training and attitudes of PE teachers. Qi and Ha (12) reviewed 75 published articles between 1990 and 2009 and generated facilitating factors for IPE, including educational stakeholders' perspectives on IPE (in- and pre-service teachers, teacher education providers, students without SEN and parents of students with SEN) and effective inclusive strategies (peer tutoring, support from paraprofessionals and PE specialists, collaborative team approach, embedded instruction, and cooperative learning). In addition, analyzing 112 articles published between 2009 and 2015, Wilhelmsen and Sørensen (13) found six main research themes guided by the approach of stakeholders, including in- and pre-service PE teachers, PE teacher educators, SEN coordinators and teaching assistants, children with and without SEN, parents and physical, educational policymakers. These systematic reviews indicate that more studies have focussed on educators' attitudes toward inclusion, particularly in Western countries (13). Qi and Ha (12) and Wilhelmsen and Sørensen (13) only included 7 and 14 studies from Asia, respectively, including only two studies conducted in 2015 from Mainland China.

### Inclusive education in china

As a part of the global movement of inclusive education, China embraced the concept of inclusive education in 1987 as the primary option for students with SEN (14). In 1988, China included inclusive education in government policy as *Learning in Regular Class* (LRC), called *Sui Ban Jiu Du* in Chinese at the national level (15). The LRC initially offered educational options to the students with three types of disabilities, including intellectual disability, visual impairment and hearing impairment in rural areas of China where few special schools and teachers were available because of limited financial resources and public transportation (15, 16). With the gradual development of the LRC model, more school-aged children with SEN enrolled in mainstream schools with their TD peers on an annual basis (17). For example, the number of students with SEN in mainstream schools increased from 129,400 in 1992 to 304,000 in the 2017 (18). These students included children with visual impairment, hearing impairment, physical disability, intellectual disability, speech disability, psychosocial disability and multi-disability (19). However, China's progress in developing inclusive education has been inadequate, and an inclusive education system has yet to be established in China (20, 21). One recent review summarized several practical problems of LRC implementation in China, including (a) inadequate funds, resources and personnel for accepting students with SEN; (b) unprepared mainstream teachers' knowledge and training in inclusive education; (c) inadequate curriculum modification; (d) TD peers' unfavorable attitudes toward students with SEN; (f) inappropriate home-school collaboration system; and (e) ineffective evaluation system on students with SEN in the regular classroom (17). Thus, there is a huge gap between the government policy on inclusive education and the current practice of LRC in China.

In China, PE is a crucial subject in the national school curriculum and is provided for students from the 1st year of primary school up to high school, including students with SEN attending mainstream schools. IPE, however, has developed slowly in China (22). A previous review of IPE in China only focused on four factors affecting IPE participation, including its regulations or policies, teachers' preparation programmes, teachers' attitudes and available curriculum and equipment (22). In addition, their review neither systemically screened articles nor was it grounded with a theoretical framework to summarize the results. Therefore, it is needed to adopt a theoretical framework to illustrate the factors affecting IPE in China.

### Socio-ecological model (SEM)

**Figure 1:**
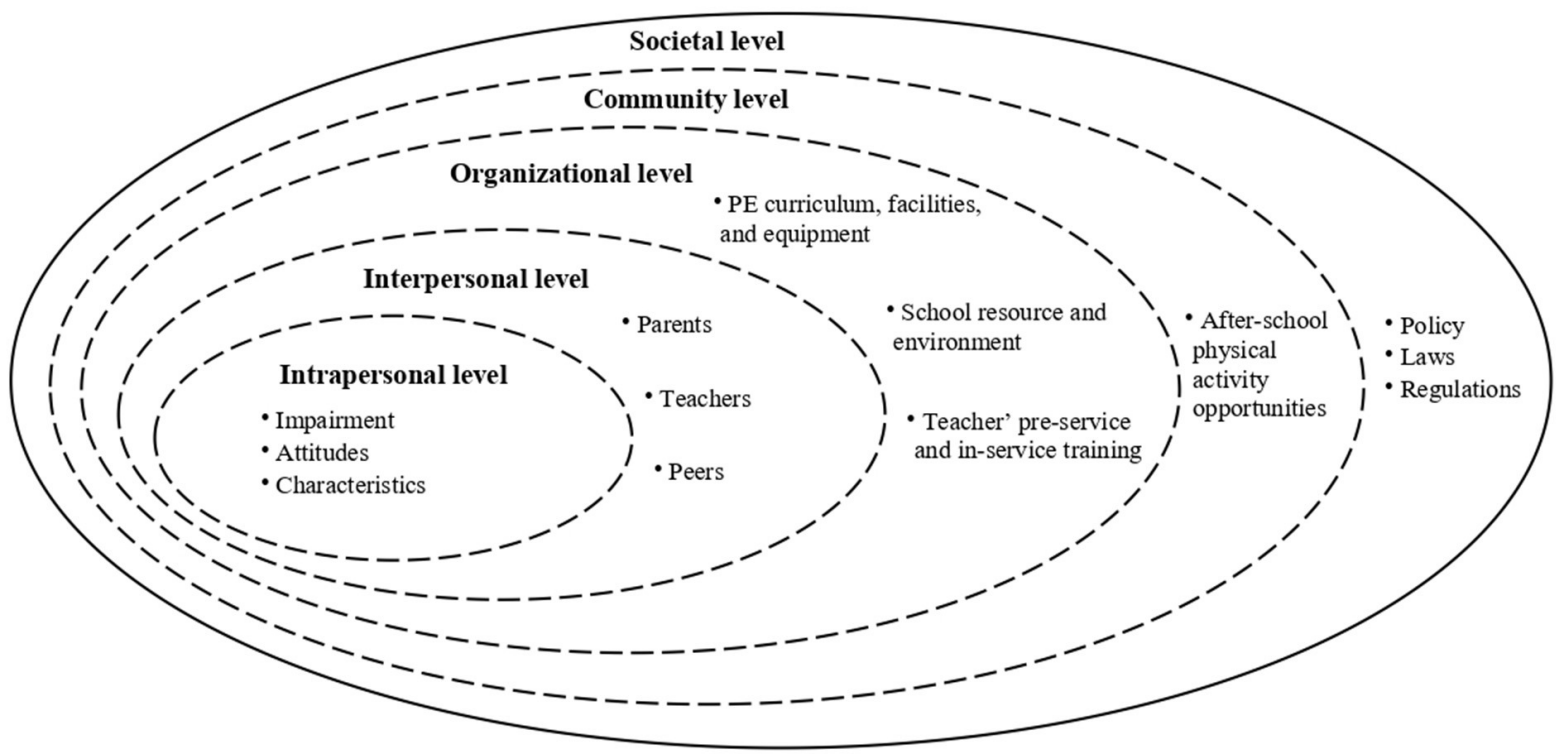
Socio-ecological model. Adapted from McLeroy et al. (23).

SEM (Figure 1) provides a theoretical framework to understand diverse factors that influence PA participation at the individual, social, and environmental levels (23). Intrapersonal factors are the center of the model that focuses on an individual's impairment, attitudes, and knowledge. The interpersonal factors are second-level factors, which refer to social relationships involving teachers, peers and family members. The third level focussed on organizational factors, such as the PE courses and PA programmes offered by schools and available PE equipment and PA facilities. Community factors are located at the fourth level, such as community-based PA programmes and extra-curricular PA opportunities offered by local PA organizations. The outermost level of SEM is the societal level, which involves public policies, laws, and regulations at various levels (23).

SEM has been widely used in various studies to identify what inhibits or facilitates participation in PE classes of individuals with and without SEN in different settings. These studies have focussed on children and adolescents with autism (24), university students with disabilities (25), children and adults with physical disabilities (26), and school PE programmes (27). Although SEM has been adopted for individuals with disabilities to understand their PA behaviors, no studies have yet applied this model to students with SEN in IPE, and to identify the factors affecting the implementation of an inclusive approach. There has been a limited review of research on IPE, specifically in China. This systematic review aimed to identify factors that affect the IPE participation of students with SEN in China at different levels using SEM. The research question guiding this review was which factors within SEM are salient in including children with SEN in IPE in China.

## Methods

### Search strategy

Electronic searches were conducted in Education Full Text (H.W. Wilson) (via EBSCOhost), SPORTDiscus with Full Text (via EBSCOhost), Eric (via EBSCOhost) and APA PsychINFO (via OVID) from inception through May 2020 and updated searching in March 2022 to identify all relevant published articles regarding the IPE in China. The search was limited to “English,” “human-related,” and “peer-reviewed” articles. The initial investigation was undertaken using three key terms: inclusive education, physical education, students with SEN. The search keywords for each primary term were developed from the search strategies of previous reviews related to inclusive PE and expert opinions in the fields of PE and inclusive education. In each database, each primary term and associated synonyms were identified, based on the following paradigm: “([Inclus^*^ OR Integration^*^ OR Exercise^*^ OR Mainstream^*^ Adapt^*^ OR Special education OR Educat^*^ OR Teach^*^ OR Learning in the regular classroom OR LRC]) AND ([Physical education OR School sport OR School-based sport OR PE]) AND ([Students with SEN OR Students with disabilit^*^ OR Students with special education needs OR Students with special needs]).” Furthermore, to include all studies related to IPE in China, location limits were not added to the screening process in four English databases. Because limited research about Chinese IPE was found through English databases, one widely used Chinese database, namely the China National Knowledge Infrastructure (CNKI), was also used in the study to search for relevant resources. The same search terms were translated to Chinese, such as “suibanjiudu,” “quannajiaoyu,” “canjirentiyu” and used in CNKI. Also, a manual search strategy was used by two independent reviewers to identify relevant articles.

### Inclusion and excuusion criteria

Studies were included if they:

- were original empirical study;
- contained descriptions of research methodology;
- focussed on the inclusion of students with SEN in IPE
- were studies conducted in Mainland China;
- were peer-reviewed articles with full-text available;
- were written in English or Chinese with English abstracts.

Studies were excluded if they:

- focused on students with disabilities in special schools;
- were literature reviews, systematic review, narrative review, case/government reports, conference papers, book chapters and validating new instruments;
- focused exclusively on PA in other environments (e.g., recess, lunchtime, after school, home);
- were studies conducted in other regions/countries rather than Mainland China.

### Data selection

**Figure 2:**
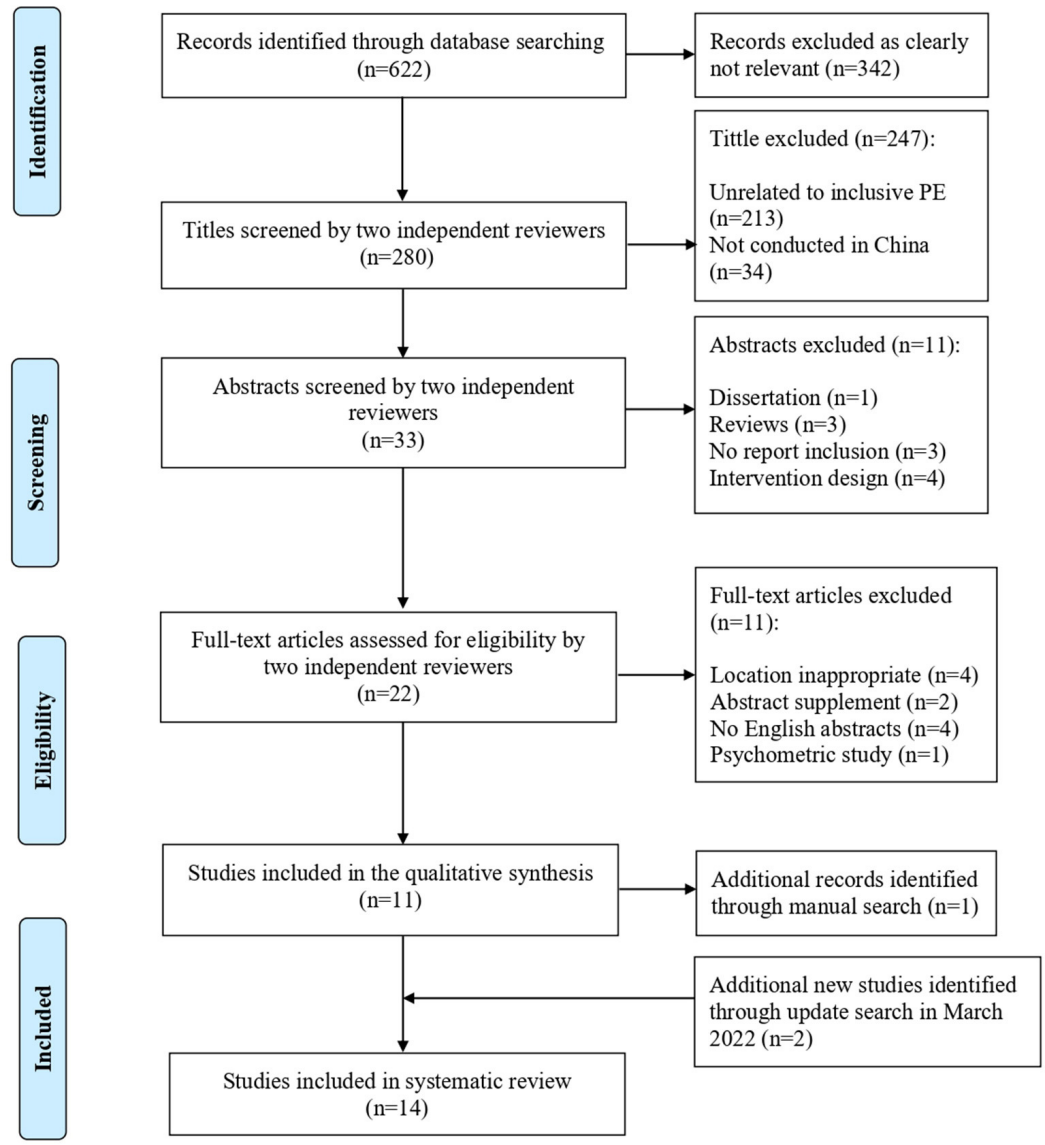
Flow diagram showing the study selection process.

A total of 622 articles were found in the initial search of the five databases described. Figure 2 illustrates the number of articles screened and those that met the inclusion criteria. To ensure the accuracy of the systematic search process, two reviewers who are familiar with the field of inclusive education and PE research independently conducted the multi-step search process. They screened the titles, abstracts, and full texts to make an initial assessment. Furthermore, if two independent reviewers disagreed with the screening papers, the third reviewer would discuss those particular papers with two reviewers and make a final call. Thirty-three abstracts met the inclusion criteria. After screening the abstracts, twenty-two articles were selected to conduct full-text screening, and 11 articles met the inclusion criteria. In addition, one manually-searched article with an agreement of two independent reviewers met the inclusion criteria. The updated search (up to March 2022) yielded an additional two studies that het the inclusion criteria. Finally, 14 articles were selected for the systematic review.

### Data extraction

Data were extracted using a standardized form, including the relevant data about bibliographic details (author and year), participant characteristics (target sample, sample size, age range, sex, school placement, location), study design, research purpose, theoretical framework, research methods, major findings, and factors related to IPE within SEM.

### Quality assessment

The McMaster Critical Review Form for quantitative and qualitative studies (28, 29) was used to evaluate the methodological quality of the included articles based on the Guidelines for Critical Review Form-Quantitative Studies and Qualitative Studies (30). The scoring criteria developed by Imms (2008) was employed to interpret the methodological quality. The three key criteria in the included quantitative studies were evaluated: sample, measurement, and analyses (31). The four key criteria in the included qualitative studies were scored: credibility, transferability, dependability, and confirmability (32). Each criterion was scored with one star (no evidence can meet any criteria); two stars (some evidence can meet the criteria, or the report is unclear); three stars (the evidence in the study can meet the criteria) (31, 32). All included studies were independently evaluated by two reviewers (XL & MH). Discrepancies between the two reviewers were resolved by discussion until consensus was finally reached.

### Data analysis

To identify factors as being “related” or “not related” to IPE participation of children with SEN, those potential factors showing a statistically significant association with the IPE participation for quantitative methods and the authors' discussion for qualitative studies were reviewed so that these could be generated and coded as IPE-related factors.

## Results

### Descriptive characteristics of included studies

A descriptive summary of the included studies is presented in Table 1. Of the 14 papers, six studies (43%) were published in peer-reviewed English journals after 2015. The included studies were mainly conducted in developed areas of China, such as Shanghai and Beijing, all of which used a cross-sectional design. In addition, 11 included studies (79%) recruited pre-and in-service PE teachers as their primary research participants, whereas four studies (33%) focussed on students with SEN, and three studies (21%) included students without SEN. Furthermore, only five studies (36%) in the English journal adopted a theoretical framework to analyse the findings; half of the Chinese journal studies adopted self-edited questionnaires and did not provide detailed information about respondents (e.g., age, sex, and educational background).

**Table 1:** Summary of participants' characteristics and quality assessment of included studies.

**Author and year**	**Participants**	**Placement**	**Data collection location**	**Sample size**	**Sex**	**Age range** **(Mean, SD)**	**Quality assessment**			
								**Sample**	**Methods**	**Analysis**
Quantitative methods										
Wu et al. (36)	The student with PD; PE teachers	University	All China	1619 students; 374 PE teachers	NR	NR	^*^	^*^	^*^	
Hao et al. (37)	In-service PE teachers, school administrators	Primary school	Beijing, Northern China	342 PE teachers; 371 school administrators	NR	21-56	**	^*^	^*^	
Han (39)	PE teachers	Primary, and secondary school	Beijing, Northern China	194	130M, 64F	21-50	**	^*^	^*^	
Liang et al. (40)	School leaders, in-service PE teachers	Primary school	Hebei Province, Northern China	65 school administrators; 72 PE teachers; 93 students with SEN	NR	NR	^*^	^*^	^*^	
Liu et al. (41)	Students with and without SEN	Primary school	Hangzhou, Southern China	60	34M, 26F	9-12	**	^*^	^*^	
Liu and Zhang (42)	Pre-service PE teachers	University	Tianjin, Wuhan, Shanghai, Guangzhou, Xi'an	1124	888M, 236F	NR (21.7, 1.6)	**	^*^	^*^	
*Wang et al. (33)	PE teachers	Primary, Secondary, High school	Shanghai, Eastern China	195	124M, 71F	22-52 (33, 6.71)	**	^*^	^*^	
Wang and Liu (45)	Pre-service PE teachers	University	Xi'an, Tianjin, Shenyang, Chengdu	644	375M, 269F	NR	^*^	^*^	^*^	
Liu and Wang (45)	Pre-service PE teachers	University	Shenyang, Chengdu, Haikou, Xiamen, Wuhan, Xi'an, Tianjin, Quanzhou	490	289M, 201F	NR	^*^	**	**	
*Wang and Qi (46)	Students with and without SEN	Primary school	Shanghai, Eastern China	872	461M, 411F	8-13 (10.79, 1.03)	**	^*^	**	
*Wang et al., (38)	Pre-service PE teachers	University	Beijing, Shenyang, Chengdu, Wuhan, Shanghai, Xiamen	490	289M, 201F	NR (21.3, 0.23)	**	^*^	**	
Qualitative methods							Credibility	Transferability	Dependability	Confirmability
*Wang et al. (33)	PE teachers	Secondary and high school	N/A	5	3M, 2F	24–55 (38.4, NR)	**	^*^	**	**
*Qi and Wang (34)	PE teachers, students with and without SEN	Secondary school	Shanghai, Eastern China	3 students with SEN; 42 students without SEN; 1 PE teacher	29M (including students with SEN and PE teacher),17F	NR	***	***	***	***
*Wang et al. (33)	Students with SEN	Secondary school	Shanghai, Eastern China	20	13M, 7F	12–16 (13, 1.13)	**	**	**	**

* English paper; M, male; F, female; SD, standard deviation; NR, not reported, no data provided; a, no criteria were met within that component; b, only some criteria were met within that component; c, all criteria were met within that component.

### Quality assessment

#### Qualitative studies

Three studies used a qualitative research design (33–35). Only one study, (34), scored the maximum ranking in all four quality assessment criteria. The study conducted data triangulation from multiple sources using multiple research methods (survey, observation, & interview). The study also provided clear and detailed information on participants' data analysis and used three layers of strategies for data trustworthiness. Two studies provided evidence to meet one or two criteria of the quality assessment (33, 35). This is because they did not report detailed information of the sample, the trustworthiness of interview data and adopted a limited method for data triangulation.

#### Quantitative studies

Eleven studies mainly used a quantitative research design (36–46). Overall, the quality of quantitative studies was lower than the qualitative studies. Few studies met all three criteria of quality assessment. All studies adopted a questionnaire as the significant approach to collect data, provided unclear information of participants and participants in all nine studies were recruited through convenience sampling. In addition, two studies (37, 40) did not report the reliability or validity of outcome measures.

### Factors affecting IPE participation of students with SEN

**Figure 3:**
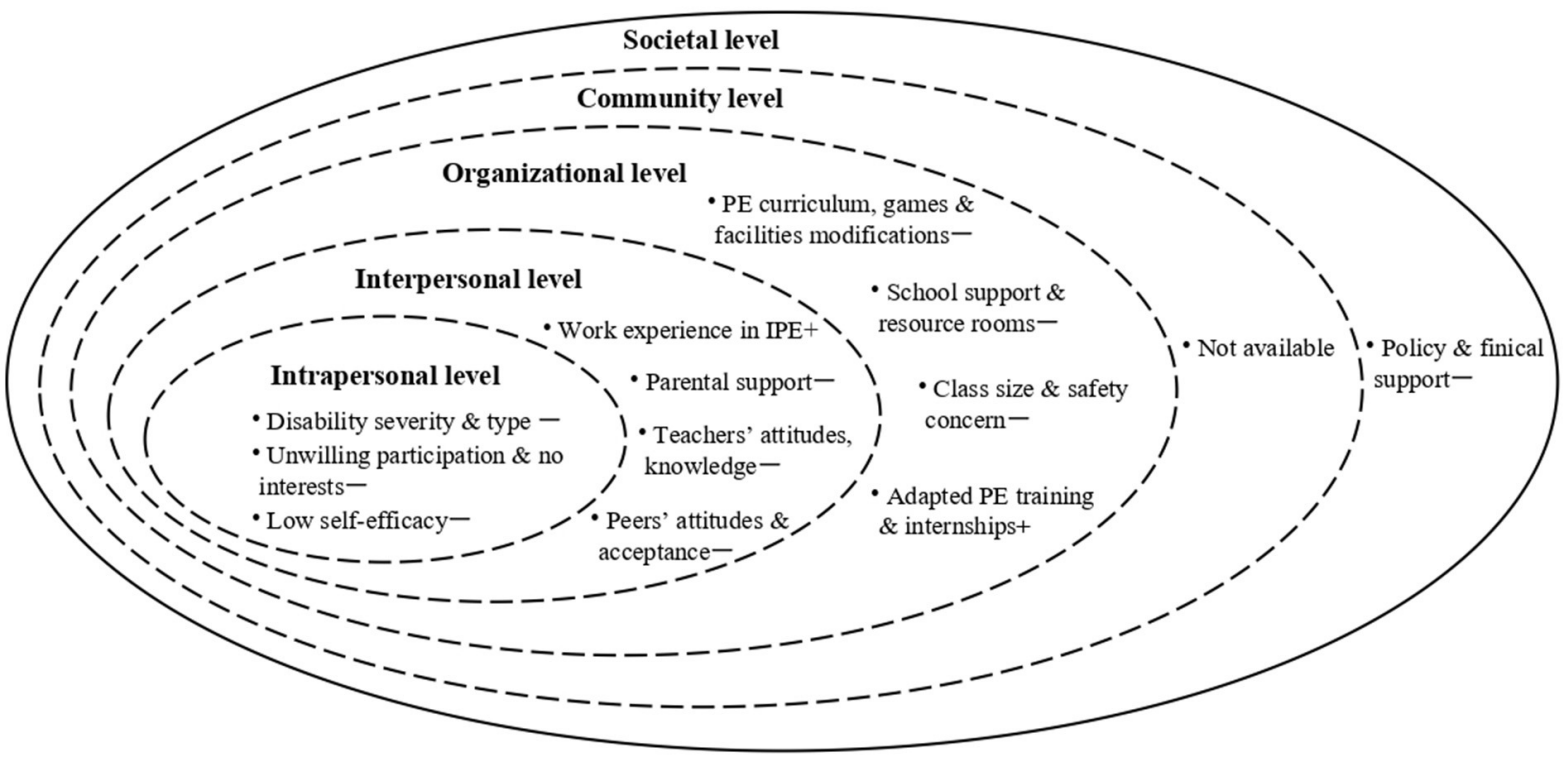
Factors affecting the IPE participation for students with SEN within the social ecological model in China. **+**, positive association; **-**, negative association.

Multi-level factors that affected the IPE participation of students with SEN within the Chinese context are summarized in Table 2. At the intrapersonal level, disability type was considered a critical factor affecting the IPE participation of students with SEN (34, 35, 39, 40, 43–45). PE educators frequently reported that students with physical disabilities had difficulty participating in IPE (35, 40, 43, 44). Also, the low self-efficacy of students with SEN decreased their interest in IPE participation (35, 40). At the interpersonal level, pre-and in-service PE teachers played a crucial role in IPE participation, including their negative attitudes toward teaching students with SEN, limited knowledge and training on adapted PE that impeded the implementation of the IPE (33, 38–40, 42, 43, 45). Besides, TD peers' negative attitudes, peer isolation and peer's perceived competitiveness also limited the IPE participation of students with SEN (34, 35, 43, 46). But, one recent study found that girls had more favorable IPE attitudes than boys (46). At the organizational level, limited school support for PE teachers and curriculum or rules modification for students with SEN hindered the IPE engagement of students with SEN (33, 35, 37, 40, 41, 43, 46). At the community level, no related research has focussed on the community-based inclusive PA programmes that affected the IPE participation of students with SEN. At the societal level, only one study reported that school leaders and PE teachers indicated that they lacked the policy and financial support to implement IPE (40). The factors affecting IPE participation in China have been summarized in Figure 3.

**Table 2:** Summary of included studies and IPE related findings.

**Author and year**	**Research** **purpose**	**Theoretical framework**	**Method**	**Major findings**	**Factors related to IPE in SEM**				
					**Individual**	**Interpersonal**	**Organisational**	**Community**	**Societal**
Wu (36)	Investigate the current situation of the acceptance of students with physical disabilities in PE class in universities	NR	Questionnaire; Interview	Nearly all surveyed students are satisfied with PE class, but PE teachers lack training and knowledge of APE to arrange diverse activities for students	NR	NR	∙ Completion of the school support system+; ∙ PE curriculum modification+	NR	NR
Hao et al. (37)	Investigate the attitudes and working condition of in-service PE teachers on LRC in Beijing	NR	Questionnaire; Interview	In-service PE teachers in Beijing hold negative attitudes to LRC and reported diverse barriers to practice	NR	NR	∙ Heavy teaching load-; ∙ Lack of resource room-	NR	NR
Han (39)	Investigate the factors that influence the attitudes toward teaching students with SEN in inclusive PE	NR	Questionnaire (PEATID- III)	Teachers who had a bachelor's degree in special education are more willing to accept students with SEN in inclusive PE.	∙ Severe disability conditions of the students-	∙ Teachers had a positive experience in teaching students SEN+; ∙ Teachers knew special education+	NR	NR	NR
Liang et al. (40)	Explore the current situation of inclusive PE in inclusive schools in Hebei Province	NR	Questionnaire	School leaders and PE teachers hold negative attitudes toward including students with SEN in general PE	∙ Students with SEN were unwilling to participate in PE-; ∙ Disability type (PD)-; ∙ Lack of participation interests-	∙ Teachers' negative attitudes toward inclusion-	∙ Lack of resource rooms-; ∙ None curriculum modifications-; ∙ Low attendance rate-	NR	∙ Lack of policy and financial support-
Liu et al. (41)	Investigate the effects of implementing inclusive PE between students with and without SEN	NR	Questionnaire	Students without SEN hold negative attitudes toward inclusive PE due to lack of professional TA during class	NR	∙ Unprepared parental involvement-	∙ Competitive PE content-; ∙ None game rules modification-; ∙ Safety concern-	NR	NR
Liu and Zhang (42)	Investigate the self-efficacy of pre-service PE teachers on teaching students with SEN in inclusive PE	NR	Questionnaire	Pre-service PE teachers showed low levels of self-efficacy in teaching students with SEN duo to lack of knowledge on APE.	NR	∙ Teachers knew APE and had internship experience+; ∙ Teachers had negative attitudes toward inclusion-	NR	NR	NR
^*^Wang et al. (43)	Examine the behavioural beliefs of PE teachers about teaching students with SEN in inclusive PE; Identify the factors that contribute to their beliefs	Behavioral Belief	Questionnaire	Beliefs of Chinese PE teachers vary according to the disability conditions. Teachers who had taken adapted-PE courses have positive beliefs about including students with SEN	∙ Disability conditions of the students (e.g. PD, emotional and behavioural problems)-	∙ Teachers had taken adapted-PE courses+; ∙ Teachers had a positive experience in teaching students SEN+; ∙ Rejection of TD peers-	∙ Lack of school support-	NR	NR
^*^Wang et al. (43)	Examines the teaching behaviour of PE teachers in teaching students with SEN; Identify factors that determine their teaching behaviours	Theory of planned behaviour	Observation; interview	PE teachers try to create a positive learning environment for students with SEN, but they lacked personnel support, failed to modify their instruction, and sometimes excluded the students with disabilities from cooperative activities	NR	∙ Teachers' positive attitudes and behavioural intention+; ∙ Lack of professional training on adapted PE-; Teachers' professional responsibilities and sense of achievement+	∙ Lack of school support-; ∙ Safety concern-; ∙ Large class size-	NR	NR
Wang and Liu (44)	Explore the self-efficacy of pre-service PE teachers who studied PE and APE toward inclusion.	NR	Questionnaire	Students who studied PE had higher self-efficacy scores than students studying APE	∙ Disability type (ID, PD, VI) -	∙ Had work experience with students with SEN00	NR	NR	NR
Liu and Wang (45)	Investigate the self-efficacy of college students who studied PE toward inclusive PE compared with students in America	NR	Questionnaire	Students who received related course knowledge in inclusive PE and work experience in students with SEN had higher self-efficacy scores	∙ Disability type (ID&PD)+	∙ Knew inclusive PE+; ∙ Had work experience with students with SEN+	NR	NR	NR
^*^Qi and Wang (34)	Examine the social interactions between students with and without SEN in inclusive PE in the Chinese context; Explore contextual factors that may determine their social interaction	The social model of disability	Observation; interview	Students with SEN have almost no social interaction with classmates without SEN in inclusive PE classes	∙ Students' disability types (e.g. autism)-	∙ TD peers' negative attitudes-	∙ Frequent individual PA programs during inclusive PE class-	NR	NR
^*^Wang (35)	Explore the perceptions of students with SEN on inclusive PE in the Chinese context; Identify the personal, physical and social context factors that facilitate or inhibit PE inclusion	Social-relational model of disability	Interview	Majority of students with special needs had negative attitudes to their inclusion, and restricted participation in physical education activities was common	∙ Disability conditions (e.g. PD) -; ∙ Low self-efficacy of students with SEN-	∙ Lack of teacher support-; ∙ Peer acceptance+ or isolation-	∙ Unprepared school environment-; ∙ Unmodified PE facilities-; ∙ Lack of curriculum & instruction modification-	NR	NR
^*^Wang and Qi (46)	Examine the general and sport-specific attitudes of elementary school students toward including students with disabilities in physical education and identifying student-related variables that determine such attitudes	NR	Questionnaire (CAIPE-R)	The Chinese students showed unfavourable general and sport-specific attitudes toward PE inclusion	NR	∙ Having a student with disabilities in PE+ ∙ Sex (i.e. female)+; ∙ Student-perceived competiviveness-	∙ Rules modification-	NR	NR
^*^Wang et al. (38)	Explore how perceived social support could affect the self-efficacy of PE major students who are expected to face students with different types of disabilities	Self-efficacy theory	Questionnaire	APE studies and internships positively affected self-efficacy among Chinese PE majors who would be facing students with different types of disabilities	NR	Perceived social support+	∙ APE courses and interships+	NR	NR

* English paper; PEATID III, Physical Educators' Attitude Toward Teaching Individuals with Disabilities III; NR, no report; +: positive association; -, negative association; 00, inconsistent association; PD, physical disability; ID, intellectual disability; VI, visual impairment; CAIPE-R, Children's Attitudes toward Integrated Physical Education – Revised Scale; APE, Adapted physical education.

## Discussion

This review aimed to explore the factors affecting IPE participation of students with SEN in China. In compliance with the SEM, the factors affecting IPE participation can be divided into five levels ranging from the intrapersonal to the societal level.

*At the intrapersonal level*, the types of disabilities of students with SEN were given more focus on inclusive PE in China. PE educators indicated that students with PD could not actively participate in IPE classes (35, 40). Early researchers in China also stated that students who suffered from severe disabilities should be taught in special schools (47). These findings are echoed by other studies in different regions and countries that a student's disability type and severity impacted IPE participation (12, 48, 49).

*At the interpersonal level*, teachers have been the primary focus. Research on attitudes toward IPE in China indicated remarkable differences between pre-and in-service teachers. For example, in-service teachers philosophically supported IPE classes in the general (33, 39, 43), but they were concerned about their limited knowledge and teaching skills and receiving insufficient support from teaching assistants and the teaching equipment (33, 40, 43). Pre-service teachers held a negative attitude toward including students with SEN in IPE classes (42, 45). This was because 64.5% of pre-service teachers had no experience in special or inclusive education courses, and only 15% of them had academic knowledge and practical experience in teaching students with SEN in IPE classes (42). Avramidis and Norwich (50) pointed out that the successful implementation of any inclusive policy is largely dependent on educators being positive about the policy. Therefore, there is a need to introduce countermeasures to help physical educators address their concerns to adopt a more positive attitude toward IPE. Secondly, pre-and in-service PE educators frequently reported that they had a difficult time providing high-quality PA programmes for all students as having limited knowledge and insufficient training or internships regarding IPE (33, 43, 45, 46). Concerning pre-service education programmes, the first teacher preparation programme for adapted PE at the undergraduate level was offered in 2001 at Tianjin University of Sports to train specialized PE teachers (22). Other adapted PE programmes at high levels have subsequently been implemented at Shandong Sport University (2004), Xi'an Physical Education University (2006), Liaoning Normal University (2006), Guangzhou Sport University (2008), Quanzhou Normal University (2009), and Wuhan Sports University (2012). In addition, Beijing Sport University (2014) and Fujian Normal University (2008) have accepted master's and doctoral students in adapted PE to cultivate professional educators. Although these universities have established programmes to accept students ranging from the undergraduate to the doctoral level, the number of graduates has been limited, and this number cannot meet the annual demands of various schools. No regular teacher training on adapted PE or IPE has been provided for in-service PE teachers regarding the in-service professional training. Moreover, the Ministry of Education (MOE) has stated that it will organize national-level training as per curriculum standards and that the Education Department of the local people's government should offer training exercises for principals and teachers in special schools and resource teachers in mainstream schools based on the newly released *Health and Physical Education Curriculum* for the blind, deaf and intellectually challenged primary and junior high school students (51). However, detailed action plans have not been published. In addition, the ‘*Special Education Promotion Plan (2017–2020)'* mentioned that professional training of no <360 h should be provided for special education teachers within 5 years (51). The MOE has launched a series of policies and plans to strengthen teacher training to promote teacher quality. Still, a regular top-down in-service training system has not been established for adapted PE or IPE teachers. Teacher education plays a key role in guiding the implementation of inclusive education by teachers (52). Brown et al. (53) also mentions that if special education-related courses are integrated into general teacher training courses, teachers who participated in such courses would have 60% more confidence to face students with SEN than students who did not receive special education training courses. Therefore, the MOE in China should organize and provide regular and systematic in-service teacher training programmes for promoting teacher professionalization. Lastly, peer rejection was also reported by PE educators and students with SEN (34, 35, 40, 43). In addition, Wang et al. (33) indicated that given the lack of professional support (teaching assistants, adapted PE specialists) in China, general PE teachers have no choice but to accept peer tutors as a Supplementary material. Qi and Wang (34) reported that students with SEN have no social interactions with their TD peers during IPE classes. In contrast, students without SEN express negative attitudes toward interacting with students with SEN during IPE classes. Peer support has been regarded as one of the key factors for implementing the IPE (54). Previous studies reveal that trained peer tutors have positive implications on IPE teaching (54, 55). Therefore, researchers and PE teachers need to design intervention programmes that focus on peer support during IPE, promoting PE participation for all students.

*At the organizational level*, a lack of support from school was one of the main barriers to inclusive PE participation. Firstly, we found that PE teachers lacked adequate professional support, such as teaching equipment, adapted physical activity specialists, resource rooms and teaching assistants within IPE settings (33, 35, 37, 40, 43). One earlier review also confirms that students with SEN received inappropriate PE services in inclusive schools due to deficiencies in support staff and facilities in the Chinese context (22). Limited equipment and teacher aides tended to be the main challenges that physical educators encountered during their daily work. Wang (35) reported that students with SEN used the same equipment as their TD peers and that the equipment size and color became an obstacle to their participation in inclusive PE.

Meanwhile, students' safety in PE classes was a significant concern for PE teachers as insufficient professional support services had been provided for teachers (33, 35). The lack of teaching assistants and education specialists substantially limits the PA participation of all children, given that the teachers have to spend time and energy ensuring the safety of students with SEN (56). Large class size has been identified as a major barrier for PE teachers in planning their classes (33). A teacher-student ratio with a class size of fewer than 30 students and one or two students with SEN within an inclusive setting is considered manageable for teachers (48, 56). Furthermore, certain PE teachers have indicated that no guidance or syllabus was provided for them to prepare for the course; thus, PE teachers had no modifications in instructions and game rules to help them include students with SEN in the IPE classes (35, 36, 40, 41). In 2007, the MOE (57) issued the *Blind School Compulsory Education Curriculum Experimental Programme*, the *Deaf School Compulsory Education Curriculum Experimental Programme* and *School for Children with intellectually challenged Compulsory Education Curriculum Experimental Programme*, which established a curriculum standard for teachers who work with children with disabilities, for reference. However, these curricula focus on Chinese, Mathematics and Life Skills, with no specific teaching guidelines for PE classes.

Moreover, with the limited participation of frontline teachers, these curricula have certain inappropriate content and have ignored the needs of students attending mainstream classes (58). In 2016, the MOE launched the latest revised version of *Compulsory Education Curriculum Standards for Deaf Schools, Compulsory Education Curriculum Standards for Blind Schools* and *Compulsory Education Curriculum Standards for Schools' Intellectually Challenged Children*. As the central area of compulsory education, *PE and Health* have been included in the curriculum standards for children with disabilities. Curriculum modification or teaching flexibility is crucial in IPE for pupils with SEN (59). The tailored teaching guidelines or curriculum standards for IPE teaching preparation can help PE educators prepare to teach content, which can promote active play for all students.

*At the community level*, we found that none of the studies have examined the effects of regular community-based PA engagement on IPE participation in students with SEN. One possible explanation is that there are a limited number of inclusive PA programmes held by PA organizations. For example, the Special Olympics was introduced in China in 1985. The Special Olympics China has organized some PA programmes for people with intellectual disabilities to promote social inclusion in collaboration with the CDPF since 1998 (60). But only 20% of participants can join in Unified Sports and most Special Olympic Programmes, which were primarily organized by special schools (60). This may explain why fewer community-based PA programmes can be introduced to promote social inclusion. Schools and parents are encouraged to arrange more PA programmes, to help students with SEN enjoy more significant social interaction with their TD peers and gain diverse experience in PA, which might lead to an interest in IPE participation with their TD peers.

*At the societal level*, one study found that PE teachers and school leaders lacked the policy and financial support to implement IPE (40). Li and Sam (22) also list some policies related to IPE to indicate that there are no specific policies or laws to support the implementation of inclusive PE. Supportive, inclusive education policy at a national level is the major driving force for ensuring the global development of inclusive education (61). Clear policies that mandate specific standards and guidelines on the time allocation of PA and PE programmes have positive implications for the promotion of PA (62). Although the Chinese government has issued LRC policies and practiced them for nearly 20 years, the contents related to IPE are limited. For example, in 2010, the Chinese government published an influential policy document, ‘*Guidelines for Mid- and Long-term Education Reform and Development (2010–2020)*'. This document positioned inclusive education as an emergent priority for education development and created governmental momentum for the inclusive education (63). Furthermore, the Chinese government has given increasing attention to ‘*Health for All*' and has issued a series of policies to implement health promotion in recent years. For example, the ‘*Health China Initiative (2019–2030)*' clearly required that primary and secondary students needed to exercise for 2 h each day, namely 1 h at school, and 1 h after school (64). More key laws and policies related to IPE in China have been summarized in Appendix. Therefore, workable policies, laws, and school regulations supporting IPE programmes, especially those focusing on inclusive PA promotion with detailed teaching assessments and guiding principles, can be expected.

This is the first systematic review to examine the factors affecting IPE participation within the Chinese context, grounded with SEM as a theoretical framework. Students with SEN have been included in IPE classes playing with their TD peers, and multi-level factors affecting their IPE engagement were identified. There are several limitations of this review. First, few studies focused on the IPE in China, and only six studies were published in English, which provided limited information to the international readers. Second, included papers emphasized the pre-and in-service PE teachers; few studies focused on the students with SEN. Although the intrapersonal level was the focus of the SEM, we could not identify enough factors at this level from the aspect of students with SEN. Thirdly, questionnaires were the most frequently used quantitative research methods in included studies, which may cause information bias or recall errors. Lastly, for studies focusing on students with SEN, interviews and observation dominated the research methods, making it challenging to understand whether students with SEN are active to meet the physical activity guidelines during the IPE classes. Therefore, objective measurement tools (i.e., accelerometer) should be considered to record PA levels of students with and without SEN during the IPE classes.

## Conclusion

We believe that the focus on IPE in China is limited. However, there is an opportunity to expand a PA promotion for students with SEN to enable them to maintain their health in inclusive settings. In addition, efforts to overcome the barriers to PA encountered by students with SEN require a comprehensive approach, especially with high-quality IPE intervention programmes. To the best of our knowledge, it is the first time that SEM has been adopted in IPE to investigate the factors that hinder or promote IPE in China. We find that the Chinese government has made great efforts to encourage the development of inclusive education and focussed more on PE and health-related programmes in recent years. However, IPE still attracts little attention from researchers. Our findings suggest that more attention and efforts to Chinese IPE development should be emphasized at organizational and community levels. From the organizational level, high-quality pre-service and in-service IPE teachers' training, IPE curriculum modification guidelines and school resource support should be provided for school IPE educators. From the community level, home-school collaboration needs to be strengthened so that parents can provide after-school PA programs and utilize community PA facilities guided by school IPE educators to help their children with SEN to be more active. Lastly, we recommend that PA and IPE researchers in China develop more tailored curriculums in IPE and provide extra-curriculum PA interventions focusing on students with SEN to help them include in the whole school.

## Data availability statement

The original contributions presented in the study are included in the article/Supplementary material, further inquiries can be directed to the corresponding author/s.

## Author contributions

XL and CS proposed and designed the review, searched, and collected the literature. XL, ML, and CS analyzed and interpreted the literature. XL drafted the manuscript. ML, YW, XW, XH, and CS contributed to the revision and approval of the submitted and final version of the manuscript. All authors contributed to the article and approved the submitted version.

## Conflict of interest

The authors declare that the research was conducted in the absence of any commercial or financial relationships that could be construed as a potential conflict of interest.

## Publisher's note

All claims expressed in this article are solely those of the authors and do not necessarily represent those of their affiliated organizations, or those of the publisher, the editors and the reviewers. Any product that may be evaluated in this article, or claim that may be made by its manufacturer, is not guaranteed or endorsed by the publisher.
