# Supplementary material for: A socio-ecological approach to inclusive physical education in China: A systematic review
Source: Front Public Health. 2022 Jul 28;10:902791. doi: 10.3389/fpubh.2022.902791 (PMC9382582; doi:10.3389/fpubh.2022.902791)
Supplement: Supplementary file 1 [file Table_1.DOCX]

*Appendix. Key Laws and Policies Related to Inclusive PE in Mainland China*

| **Year** | **Policy or law** | **Content related to inclusive PE** | **Policy publisher** |
| --- | --- | --- | --- |
| 1986 | Compulsory Education Law of the People’s Republic of China | Local people's government should establish special schools (or classes) for children and adolescents who are blind, deaf mute or retarded to receive compulsory education. | National People’s Congress of the People’s Republic of China |
| 1991 | Law of the People’s Republic of China on the Protection of Disabled Persons | The state and society should encourage and assist disabled persons to participate in various cultural, sports and recreational activities and strive to meet the needs of the spiritual and cultural life of disabled persons. | National People’s Congress of the People’s Republic of China |
| 1994 | Regulations on Education for Persons with Disabilities | The Education Department of the local people’s government should develop the policy of ‘Learning in a Regular Classroom’ (LRC), and schools must provide regular physical settings for students with disabilities. | State Council of the People’s Republic of China |
| 1995 | Law of the People’s Republic of China on Physical Culture and Sports | Schools should organise suitable sports activities for students with disabilities to meet their diverse needs. | National People’s Congress of the People’s Republic of China |
| 2006 | Eleventh Five-Year Program - Outline for the Development of the Cause of Disabled Persons | The state implements the National Fitness Program and supports disabled people to participate in physical activities. In addition, normal and sports universities should cultivate qualified teachers and enrol students with disabilities in higher education. | State Council of the People’s Republic of China |
| 2008 | Opinions of the CPC Central Committee and the State Council on Promoting the Development of the Cause of Disabled Persons (No.7 [2008] of the CPC Central Committee) | Normal and sports universities should conduct research on adapted PE and develop the subject for students with disabilities. The local people's government at various levels should arrange for individuals with disabilities to actively participate in the Para Olympics, Special Olympics and Deaf Olympics. | State Council of the People’s Republic of China |
| 2009 | Regulation on National Fitness | In the formulation of a national fitness plan or implementation plan, full consideration must be given to the special needs of students, seniors, the disabled and rural residents. | State Council of the People’s Republic of China |
| 2011 | National Fitness Plan (2011–2015) | Adapted PE must be strongly promoted. All special and mainstream schools should pay attention to PE and provide appropriate physical fitness and physical rehabilitation programmes for students with disabilities. | State Council of the People’s Republic of China |
| 2014 | Special Education Promotion Plan (2014–2016) | Mainstream schools should increase the quota of LRC and promote teacher training to improve the quality of inclusive education. | Ministry of Education of the People’s Republic of China |
| 2016 | Opinions on Strengthening School Physical Education and Promoting Comprehensive Development of Students’ Physical and Mental Health | Researching and promoting PE resources which are suitable for different types of disabilities, improving the quality of PE for students with disabilities and ensuring that each student has the right to receive PE | General Office of the State Council of the People’s Republic of China |
| 2016 | Outline of the Healthy China 2030 Plan | Students should spend no less than one hour doing sports exercises on campus every day. The configuration rate of school sports facilities and equipment will reach 100% by 2030, and students will participate in MVPA for more than three times per week. The development of rehabilitation and fitness sports for individuals with disabilities must be promoted. | General Office of the State Council of the People’s Republic of China |
| 2016 | Measurement Plan for Disabled Persons on Physical Culture and Sports during the ‘13th Five-Year Plan’ period | The state encourages students from high schools, primary schools and universities to participate in the Special Olympics University Program and inclusive physical activities. Diverse special and mainstream schools must organise daily regular physical activities, develop after-school sports activities and conduct inclusive school activities for students with disabilities considering their own characteristics. | China Disabled Persons’ Federation  & General Administration of Sport of China |
| 2017 | Regulations on the Work Concerning Physical Education and Sport in Schools (2017 Revision) | Ordinary primary and secondary schools should set up exercises during break time every day and more than three extracurricular sports activities every week to ensure that students have one hour of physical activities daily (including PE classes). | State Council of the People’s Republic of China |
| 2017 | Regulation on the Education of the Disabled (2017 Revision) | The quality of education for the disabled must be improved. Inclusive education should be actively promoted. Normal and special education methods must be adopted in accordance with the types of disabilities and learning capacities of the disabled. The adoption of the normal education method should be prioritised. | General Office of the State Council of the People’s Republic of China |
| 2018 | National Student Physical Health Standard | Evaluation indicators are set for students of full-time ordinary elementary schools, junior high schools, ordinary high schools, secondary vocational schools and ordinary higher education institutions. | Ministry of Education of the People’s Republic of China |
| 2019 | China's Education Modernization 2035 | The government should highlight the special schooling for the disabled and comprehensively promote inclusive education. | General Office of the State Council of the People’s Republic of China |
